# Mental Health of Nursing Students amid Coronavirus Disease 2019 Pandemic

**DOI:** 10.3389/fpsyg.2021.699558

**Published:** 2021-08-12

**Authors:** Juan Gao, Fengyun Wang, Shengcun Guo, Fudong Hu

**Affiliations:** ^1^School of Nursing, Henan Technical Institute, Zhengzhou, China; ^2^Department of Cardiology, The First Affiliated Hospital of Zhengzhou University, Zhengzhou, China

**Keywords:** mental health, PTSD, depression, anxiety, stress, nursing students, COVID-19

## Abstract

The coronavirus disease 2019 (COVID-19) pandemic is a global disaster, and recent studies have shown its association with increasing mental health problems such as post-traumatic stress disorder (PTSD), depression, anxiety, and stress. Nursing students, especially nursing interns, may be shunned, harassed, and even blamed as potential COVID-19 spreaders, though they were an important reserve force against COVID-19 and other diseases. Of note, the psychological influences of COVID-19 on nursing students remained unclear. The aim of this study was to evaluate the mental health of nursing students during the COVID-19 pandemic. A cross-sectional online survey was conducted on nursing students in a vocational college from April 12 to 23, 2020. The Impact of Event Scale–Revised, 21-item Depression, Anxiety and Stress Scale, and Pittsburgh Sleep Quality Index were used to assess the degree of symptoms of PTSD, depression, anxiety, stress, and insomnia, respectively. Multivariable logistic regression analysis was performed to determine the potential risk factors for the psychological symptoms. A total of 1,780 college nursing students were asked to participate in this online survey, with 1,532 complete responses. In total, 682 (44.5%) college nursing students reported having PTSD, 358 (22.8%) students reported insomnia, and few students reported depression (*n* = 45, 2.9%), anxiety (*n* = 44, 2.9%), and stress (*n* = 17, 1.1%) symptoms. As compared with junior, female, and rural nursing students, the senior, male, and urban nursing students had higher rates of PTSD, depression, anxiety, stress, respectively, whereas male nursing students had a higher insomnia rate. Multivariable analysis showed that senior nursing students had higher risks of PTSD, depression, anxiety; being male was associated with higher risks of PTSD, depression, anxiety, stress, and insomnia; and urban nursing students had higher risks of PTSD, depression, anxiety, and stress. In summary, a considerable number of nursing students reported mental symptoms of PTSD and insomnia, though few reported mental symptoms of depression, anxiety, and stress. Furthermore, senior, male, and urban nursing students are at risk for developing mental symptoms. Appropriate psychological interventions should be implemented to assure the mental health of nursing students.

## Introduction

The coronavirus disease 2019 (COVID-19) was first identified in Wuhan, China on December 8, 2019, and it has rapidly spread all over the world (Pan et al., 2020). This pandemic has been a global disaster, greatly influencing social lives, economics, mental conditions, and health security, and it seemed that there was no end to this disaster (Firth et al., 2020; Pfefferbaum and North, 2020). As of June 4, 2021 (the moment of writing), WHO reported 171,782,908 confirmed cases of COVID-19 globally, with 3,698,621 deaths (World Health Organization, 2021).

To limit the spread of this outbreak, a series of important and effective public-health measures were implemented in China and in many other countries, such as mass vaccination, lockdowns, maintaining social distancing, wearing masks, hand hygiene, population surveillance, abundant COVID-19 tests, rigorous contacts tracing, mandatory quarantine for confirmed or suspected cases with COVID-19 infection and their close contacts, and the building of makeshift hospitals (Budd et al., 2020; Firth et al., 2020; Pan et al., 2020; Ruktanonchai et al., 2020). Despite the initiation of these public-health measures, cases continued to rise. Many infected patients had returned to China from other countries, and imported infections as well as asymptomatic cases were the main challenges in China (Chen et al., 2020). In order to curb the spread of COVID-19, Chinese students had experienced prolonged school suspensions and subsequent online education at home.

During the initial stage of the COVID-19 epidemic, the results of a survey conducted among the general population in China had shown that more than half of the respondents (53.8%) rated the psychological impact of the COVID-19 epidemic as moderate to severe (Wang X. et al., 2020). With the increasing mental health burden amid the COVID-19 pandemic, it became crucial and necessary to enhance mental health assessments and support so as to maintain public mental health, and the Chinese National Health Commission has issued guidelines for emergency psychological crisis intervention and established psychological assistance hotlines (Lai et al., 2020; Wang Y. et al., 2020). Many mental healthcare workers have been bravely and voluntarily involved in providing frontline or online psychological care (e.g., WeChat, TikTok, Weibo, and hotlines) to patients with COVID-19 as well as to the general population (Wang Y. et al., 2020). These mental health services might improve the mental resilience and reduce the incidence of psychological diseases. However, several people have not requested for mental health assistance due to the fear of discrimination and stigmatization, despite having severe psychological symptoms (Lyndon et al., 2019; Javed et al., 2021).

Healthcare workers, including nurses, played very important roles in fighting the COVID-19 pandemic and have made invaluable contributions. Many nurses were brave and heroic, working at the frontlines to treat patients with COVID-19 pneumonia despite the very high risk of infection (Hartmann et al., 2020; Hughes et al., 2020). Recent studies have shown that healthcare workers such as frontline nurses, especially women, experienced a large psychological burden which manifested as symptoms of post-traumatic stress disorder (PTSD), depression, anxiety, and insomnia (Lai et al., 2020).

Some studies had shown that the psychological consequences of the COVID-19 epidemic on college students could be serious. Chinese college students suffered from symptoms of PTSD, stress, anxiety, and depression in the early stage of the COVID-19 pandemic (Li H. Y. et al., 2020; Tang et al., 2020; Zhang et al., 2020). In addition, medical students may suffer from more stress than non-medical students following the COVID-19 outbreak (Ye et al., 2020).

Nursing students are a new and important reserve force against COVID-19 and other diseases. In China, nursing students in general are required to receive 3–5 years of a college education. They also need to undergo clinical training as interns in the hospital during their previous year. Later, some nursing students continue further studies, whereas most nursing students begin working as formal clinical nurses after graduation.

At the early beginning of the COVID-19 epidemic, though WHO was informed of a cluster of pneumonia of unknown cause in Wuhan city on December 31, 2019 (World Health Organization, 2020), much remained unknown except for patients with COVID-19 were infected by direct exposure at the seafood market (The Lancet, 2020b), and Chinese nursing students were still learning at schools or hospitals during this period. On January 20, 2020, the eminent SARS specialist Zhong Nanshan announced that 14 medical workers had been infected by one virus carrier and confirmed that COVID-19 could spread from human to human (Nature, 2020). Also on January 20, the Chinese president and government announced the COVID-19 outbreak and it should be resolutely contained (Nature, 2020). At this time, most junior nursing students had been back home to celebrate Chinese New Year, but nursing seniors (interns) still remained in their clinical training in the hospital. Massive actions including all sectors from business to factories and to schools were taken the next day to curb the COVID-19 epidemic (Chen and Yu, 2020). As of January 23, 2020, a total of 835 confirmed cases (549 from Hubei Province and 286 in 32 provinces, municipalities, and special administrative regions in China) were detected; in order to contain the COVID-19 spread, Wuhan City was locked down, and soon followed by many other areas in China (The Lancet, 2020b; Wang et al., 2020a,b). Of note, as cases increased, medical workers were recognized as a high-risk group to acquire the COVID-19 infection. As of February 11, 2020, a total of 72,314 COVID-19 cases were reported in mainland China, with 3,019 medical workers (1,716 were confirmed) (Epidemiology Working Group for NCIP Epidemic Response Chinese Center for Disease Control Prevention, 2020). Since senior nursing students needed to work as interns in the hospital before Spring Festival, they were considered as medical workers by the general population. The public, including friends and relatives, had a fear of getting COVID-19 infection, so as to the medical workers such as nurses even nursing students were shunned, harassed, and even blamed as potential COVID-19 spreaders by some people (Bagcchi, 2020; Koh, 2020; Abdulah et al., 2021). Besides, the nursing students who worked in the hospital were also anxious about the chance of getting the COVID-19 infection and passing the infection to their families (The Lancet, 2020a). Nursing students experienced extreme psychological stress and a range of feelings such as excitement, doubt, and helplessness after the COVID-19 outbreak (Huang et al., 2020).

To date, it is still unclear whether the COVID-19 pandemic and the subsequent quarantine and online education could give rise to mental health symptoms among college nursing students. According to the official website, from April 12 to 23, 2020, there were 1,273 COVID-19-confirmed patients in the Henan Province (with no new increases), and confirmed cases in China increased from 83,597 to 84,303 (moving into mitigation stage), whereas confirmed cases all over the world rapidly increased from 1,713,517 to 2,548,755 (World Health Organization, 2021). During this period, we conducted a survey that focused on the mental health (including symptoms of PTSD, depression, anxiety, stress, and insomnia) among nursing students studying in Henan Technical Institute, a comprehensive vocational college with a 3-year nursing college education with almost 2,200 nursing students located in Zhengzhou, Henan Province, China, in order to provide evidence for the formation of specific and effective mental health interventions for nursing students.

## Materials and Methods

### Study Design and Participants

A cross-sectional online survey regarding the mental health of college nursing students was conducted from April 12 to 23, 2020 according to the principles of the Declaration of Helsinki. Most nursing students (*n* = 1,780) in Henan Technical Institute were asked to voluntarily participate in this study by their teachers via QQ groups (a widely used instant messaging and social platform, Tencent Inc., Shenzhen, China), and all the participants were informed that they had the right to terminate their participation in the study anytime they desired. All these students received online education at home in order to avoid infection with COVID-19, hence the survey was completed *via* an online platform (SurveyStar, a professional online survey, examination, and voting platform, Changsha Ranxing Information Technology Co., LTD, Shanghai, China), especially WeChat/Weixin (a widely used communication and social platform in China, Tencent Inc., Shenzhen, China), using a cellphone or computer. The questions of this survey could be revisited and answered using the same WeChat account, but they could not be corrected or answered again once these were submitted. Students who responded were divided into two groups according to their year level: 1,135 were junior nursing students (grades 1 and 2) and 397 were senior nursing interns (grade 3).

Participation in this study was anonymous and the personal information of participants was kept confidential. This study protocol was approved by the Ethics Committee of Henan Technical Institute.

### Measurements

The mental health conditions of nursing students amid the COVID-19 pandemic were evaluated through an online structured questionnaire. The 22-item Impact of Event Scale–Revised (IES-R) (Christianson and Marren, 2012), 21-item Depression, Anxiety, and Stress Scale (DASS-21) (Lovibond and Lovibond, 1995; Henry and Crawford, 2005), and Pittsburgh Sleep Quality Index (PSQI) (Buysse et al., 1989) were used to assess the symptoms of PTSD, depression, anxiety, stress, and insomnia among college nursing students, respectively. IES-R included intrusion (items of IES-R 1, 2, 3, 6, 9, 14, 16, 20), avoidance (items 5, 7, 8, 11, 12, 13, 17, 22), and hyperarousal (items 4, 10, 15, 18, 19, 21) subscales, with five choices including not at all (score 0), a little bit (score 1), moderately (score 2), quite a bit (score 3), and extremely (score 4). The severity of PTSD symptoms was evaluated by the sum of the intrusion and avoidance subscales (Christianson and Marren, 2012). The DASS-21 included depression (items of DASS 3, 5, 10, 13, 16, 17, 21), anxiety (items 2, 4, 7, 9, 15, 19, 20), and stress (items 6, 8, 11, 12, 14, 18) subscales, with four responses including do not apply to me at all (score 0), apply to me to some degree or some of the time (score 1), apply to me a considerable degree or a good part of the time (score 2), and apply to me very much or most of the time (score 3). The final score of each subscale was equal to the sum of its items and then multiplied by two, as the DASS-21 was a short form version of the 42-item DASS (Lovibond and Lovibond, 1995; Henry and Crawford, 2005). The PSQI evaluated seven components of sleep quality, sleep latency, sleep duration, sleep efficiency, sleep disturbance, use of sleeping medication, and daytime dysfunction. The score ranged from 0 to 3 for each component, and the PSQI score was equal to the sum of the seven component scores (Buysse et al., 1989). The detailed questions and scoring methods are shown in Supplementary Material. The scales above have been shown to have excellent reliability and validity in previous studies (Yohannes et al., 2019; Chew et al., 2020; Lai et al., 2020; Xiao et al., 2020).

The scores of the above scales were graded as follows: scores on the IES-R (sum of intrusion and avoidance subscale scores) were classified as normal (0–8), mild (9–25), moderate (26–43), and severe (44–64) PTSD (Christianson and Marren, 2012; Lai et al., 2020). For the DASS-21 depression subscales, participants were classified as being normal (0–9) or having mild (10–13), moderate (14–20), severe (21–27), or extremely severe (28–42) depression. For the DASS-21 anxiety subscale, classifications included normal (0–7), mild (8–9), moderate (10–14), severe (15–19), and extremely severe (20–42) anxiety. For the DASS-21 stress subscale, classifications included normal (0–14), mild (15–18), moderate (19–25), severe (26–33), and extremely severe (34–42) stress (Lovibond and Lovibond, 1995; Henry and Crawford, 2005; Yohannes et al., 2019; Chew et al., 2020). Finally, the PSQI included categories such as normal (0–5), mild (6–10), moderate (11–15), and severe (16–21) insomnia (Buysse et al., 1989; Xiao et al., 2020).

In addition, the educational and living conditions of nursing students during the COVID-19 pandemic were also inquired about in this survey. There were three questions related to their education: (1) attitude toward online education, with four responses including very satisfactory, satisfactory, average, or unsatisfactory; (2) mental states during online learning, with three responses including better, as usual, or worse; and (3) attitude toward going back to school, with two responses including expected and not expected. There were five questions related to their living conditions: (1) family economic income (higher, as usual, or lower); (2) body weight (increased, unchanged, or decreased); (3) quality of life (good, average, or bad); (4) attention to COVID-19 (always, usually, sometimes, or almost never); and (5) attitude toward being a frontline nurse (sure, maybe, maybe not, or impossible).

### Covariates

Demographic data such as gender (male or female), educational status (junior or senior), and place of residence (urban or rural) were collected in the survey. The COVID-19 status of the nursing students and their families was also investigated.

### Statistical Methods

All statistical tests were performed using SPSS software (version 22.0, SPSS, IBM Corporation, Armonk, New York). Scores of measurement scales had a skewed distribution, and these were presented as medians and interquartile ranges (IQR). A non-parametric Mann–Whitney *U*-test was used to compare the differences between the two groups. Categorical variables, such as the severity classifications of PTSD, depression, anxiety, stress, and insomnia symptoms, were presented as numbers and percentages, and group differences were assessed using the Mann–Whitney *U*-test of ranked data.

Multivariable logistic regression analysis was applied to evaluate the influences of educational status, gender, and location on PTSD, depression, anxiety, stress, and insomnia symptoms, and their associations were demonstrated as OR with a 95% CI.

A *P* < 0.05 was considered significant, and all tests were two-tailed.

## Results

### Demographic Characteristics

In this study, a total of 1,780 college nursing students were asked to fulfill the online survey (the count included QQ groups of teachers who asked their students and sent the link to the survey), and we received 1,532 complete responses, with a response rate of 86.1%. Among these 1,532 nursing students, the average age was 19.95 (SD, 1.24) years, 397 (25.9%) were senior interns, 1,135 (74.1%) were juniors, 666 (43.5%) came from an urban area, 866 (56.5%) came from a rural area, and 1,400 (91.4%) lived in the Henan Province. The majority of respondents were females (*n* = 1,351, 88.2%), with 181 (11.8%) males. There were no confirmed or suspected COVID-19 infected cases in this research according to self-reports and official data from the school.

### The Educational and Living Conditions Influenced by COVID-19

Table 1 presents the educational and living conditions of respondents. As for educational condition, many college nursing students were satisfied with their current online education (satisfactory, *n* = 1,036, 67.6%; very satisfactory, *n* = 234, 15.3%). However, only 164 (10.7%) respondents reported having better mental states during online learning, whereas many students (*n* = 883, 57.6%) reported worsening mental states and 1,359 (88.7%) students expected to go back to school. As for their living condition, 1,261 (82.3%) suffered a loss in their family income, 265 (17.3%) reported the same economic income as usual, and only 6 (0.4%) reported a higher economic income. Surprisingly, only 52 (3.4%) students thought they had a poor quality of life. Furthermore, 675 (44.1%) students had gained weight. Of the 1,532 respondents, 696 (45.4%) reported that they usually paid attention to the COVID-19 pandemic, and 633 (41.3%) reported always paying close attention to COVID-19. Finally, an overwhelming majority of students had a positive attitude (*n* = 1,504, 98.2%) toward becoming frontline nurses against COVID-19 in the future.

**Table 1:** Educational and living conditions of all and subgroup participants.

	**Total**	**Educational status**				**Gender**				**Location**			
**Variables**	***N*** **= 1,532**	**Junior** ***N*** **= 1,135**	**Senior** ***N*** **= 397**	***Z***	***P***	**Female** ***N*** **= 1,351**	**Male** ***N*** **= 181**	***Z***	***P***	**Rural** ***N*** **= 866**	**Urban** ***N*** **= 666**	***Z***	***P***
**Education**													
Attitude toward online education				1.721	0.085			0.455	0.649			1.925	0.054
Very satisfactory	234 (15.3)	158 (13.9)	76 (19.1)			201 (14.9)	33 (18.2)			115 (13.3)	119 (17.9)		
Satisfactory	1,036 (67.6)	781 (68.8)	255 (64.2)			927 (68.6)	109 (60.2)			599 (69.2)	437 (65.6)		
Average	216 (14.1)	163 (14.4)	53 (13.4)			186 (13.8)	30 (16.6)			124 (14.3)	92 (13.8)		
Unsatisfactory	46 (3%)	33 (2.9)	13 (3.3)			37 (2.7)	9 (5.0)			28 (3.2)	18 (2.7)		
Mental states during online learning				2.797	0.005			2.453	0.014			0.911	0.362
Better	164 (10.7)	112 (9.9)	52 (13.1)			153 (11.3)	11 (6.1)			101 (11.7)	63 (9.5)		
As usual	485 (31.7)	346 (30.5)	139 (35.0)			433 (32.1)	52 (28.7)			272 (31.4)	213 (32.0)		
Worse	883 (57.6)	677 (59.6)	206 (51.9)			765 (56.6)	118 (65.2)			493 (56.9)	390 (58.6)		
Attitude toward going back to school				0.153	0.878			2.860	0.004			0.943	0.346
Expected	1,359 (88.7)	1,006 (88.6)	353 (88.9)			1,187 (87.9)	172 (95.0)			774 (89.4)	585 (87.8)		
Not expected	173 (11.3)	129 (11.4)	44 (11.1)			164 (12.1)	9 (5.0)			92 (10.6)	81 (12.2)		
**Living**													
Family economic incomes				2.635	0.008			1.562	0.118			2.858	0.004
Higher	6 (0.4)	5 (0.4)	1 (0.3)			2 (0.1)	4 (2.2)			3 (0.3)	3 (0.5)		
As usual	265 (17.3)	213 (18.8)	52 (13.1)			230 (17.0)	35 (19.3)			129 (14.9)	136 (20.4)		
Lower	1,261 (82.3)	917 (80.8)	344 (86.6)			1,119 (82.8)	142 (78.5)			734 (84.8)	527 (79.1)		
Body weight				0.296	0.767			0.953	0.341			0.665	0.506
Increased	675 (44.1)	501 (44.1)	174 (43.8)			590 (43.7)	85 (47.0)			383 (44.2)	292 (43.8)		
Unchanged	725 (47.3)	530 (46.7)	195 (49.1)			642 (47.5)	83 (45.9)			418 (48.3)	307 (46.1)		
Decreased	132 (8.6)	104 (9.2)	28 (7.1)			119 (8.8)	13 (7.2)			65 (7.5)	67 (10.1)		
Quality of life				1.497	0.134			0.952	0.341			0.873	0.382
Good	897 (58.6)	678 (59.7)	219 (55.2)			796 (58.9)	101 (55.8)			499 (57.6)	398 (59.8)		
Average	583 (38.1)	418 (36.8)	165 (41.6)			512 (37.9)	71 (39.2)			336 (38.8)	247 (37.1)		
Bad	52 (3.4)	39 (3.4)	13 (3.3)			43 (3.2)	9 (5.0)			31 (3.6)	21 (3.2)		
Attention to COVID-19				2.866	0.004			2.683	0.007			2.558	0.011
Always	633 (41.3)	449 (39.6)	184 (46.3)			541 (40.0)	92 (50.8)			336 (38.8)	297 (44.6)		
Usually	696 (45.4)	521 (45.9)	175 (44.1)			626 (46.3)	70 (38.7)			403 (46.5)	293 (44.0)		
Sometimes	202 (13.2)	164 (14.4)	38 (9.6)			183 (13.5)	19 (10.5)			126 (14.5)	76 (11.4)		
Almost never	1 (0.1)	1 (0.1)	0 (0.0)			1 (0.1)	0 (0.0)			1 (0.1)	0 (0.0)		
Attitude toward being frontline nurse				2.788	0.005			2.917	0.004			0.181	0.857
Sure	1,043 (68.1)	750 (66.1)	293 (73.8)			901 (66.7)	142 (78.5)			591 (68.2)	452 (67.9)		
May be	461 (30.1)	364 (32.1)	97 (24.4)			429 (31.8)	32 (17.7)			260 (30.0)	201 (30.2)		
May be not	24 (1.6)	18 (1.6)	6 (1.5)			18 (1.3)	6 (3.3)			12 (1.4)	12 (1.8)		
Impossible	4 (0.3)	3 (0.3)	1 (0.3)			3 (0.2)	1 (0.6)			3 (0.3)	1 (0.2)		

On comparing junior and senior nursing students, senior nursing students (interns) reported having better mental states during the online learning period (worse, 51.9 vs. 59.6%, *P* < 0.01) but worse family economic incomes (lower, 86.6 vs. 80.8%, *P* < 0.01). They also paid more attention to COVID-19 (always and usually, 90.4 vs. 85.5%, *P* < 0.01) and were more willing to become frontline nurses against COVID-19 (sure, 73.8 vs. 66.1%, *P* < 0.01). When compared with female nursing students, male nursing students also reported paying more attention to COVID-19 (always and usually, 89.5 vs. 86.3%, *P* < 0.01) and being more willing toward becoming frontline nurses against COVID-19 (sure, 78.5 vs. 66.7%, *P* < 0.01). However, they reported worse mental states during the online learning period (worse, 65.2 vs. 56.6%, *P* < 0.05). There were no significant differences in changes in family economic income according to sex. With respect to the rate of nursing students who expected to go back to school, this was higher among males than females (95 vs. 87.9%, *P* < 0.01), but there was no significant difference between senior and junior nursing students. There were no significant differences between junior and senior nursing students in terms of their degree of satisfaction with online education, changes in body weight, and quality of life.

With regard to rural and urban nursing students, urban nursing students reported better family economic incomes (lower, 79.1 vs. 84.8%, *P* < 0.01) and they paid more attention to COVID-19 (always and usually, 88.6 vs. 85.3%, *P* < 0.05) as compared with rural nursing students; however, there were no significant differences in all other educational and living indexes.

### Mental Health Outcomes

**Figure 1:**
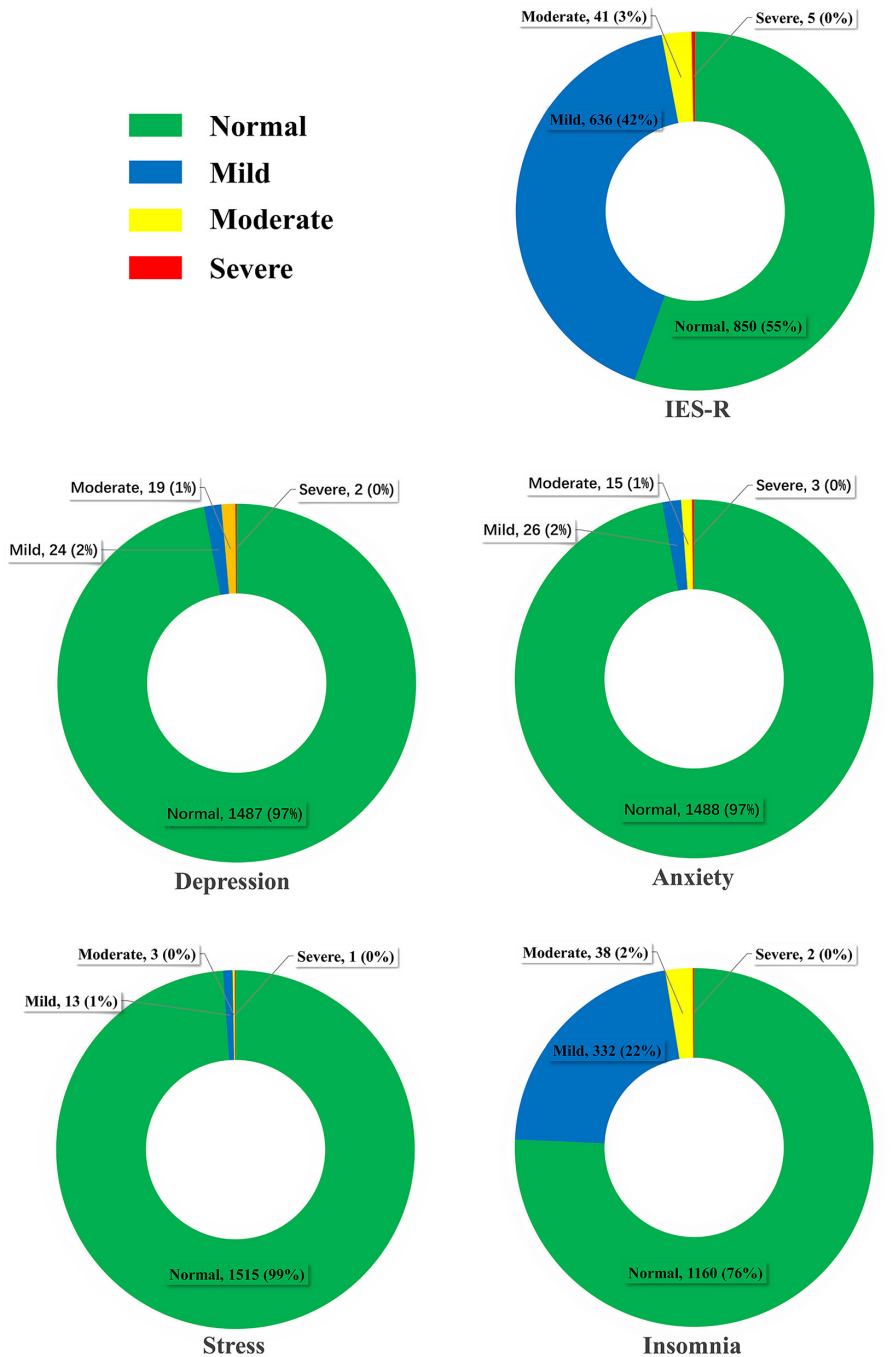
Severity classifications of post-traumatic stress disorder, depression, anxiety, stress, and insomnia in nursing students.

As shown in Table 2, the median (IQR) score of IES-R for PTSD was 7 (3–14), the median (IQR) scores of DASS-21 for depression, anxiety, and stress were 0 (0–2), 0 (0–2), and 1 (0–3), respectively, and the median (IQR) score of PSQI for insomnia was 3 (2–5). As shown in Table 3 and Figure 1, a considerable number of college nursing students (*n* = 682, 44.5%) reported having symptoms of PTSD, few students reported symptoms of depression (*n* = 45, 2.9%), anxiety, (*n* = 44, 2.9%), and stress (*n* = 17, 1.1%), whereas the number of students with insomnia (*n* = 358, 22.8%) was also considerable. Among these students, only a few experienced symptoms of severe PTSD (*n* = 5, 0.3%), depression (*n* = 2, 0.1%), anxiety (*n* = 3, 0.2%), stress (*n* = 1, 0.1%), and insomnia (*n* = 2, 0.1%).

**Table 2:** Scores of PTSD, depression, anxiety, stress, and insomnia.

	**Total**	**Educational status**				**Gender**				**Location**			
**Scales**	***N*** **= 1,532**	**Junior** ***N*** **= 1,135**	**Senior (interns)** ***N*** **= 397**	***Z***	***P***	**Female** ***N*** **= 1,351**	**Male** ***N*** **= 181**	***Z***	***P***	**Rural** ***N*** **= 866**	**Urban** ***N*** **= 666**	***Z***	***P***
IES-R, PTSD	7 (3–14)	7 (3–14)	9 (3–15)	2.714	0.007	7 (3–14)	11 (3–16)	2.830	0.005	7 (3–13)	8 (3–15)	2.512	0.012
DASS, depression	0 (0–2)	0 (0–2)	1 (0–4)	4.151	<0.001	0 (0–2)	1 (0–5)	3.358	0.001	0 (0–2)	0 (0–3)	1.798	0.072
DASS, anxiety	0 (0–2)	0 (0–1)	0 (0–3)	2.591	0.010	0 (0–2)	0 (0–3)	3.613	<0.001	0 (0–1)	0 (0–2)	2.412	0.016
DASS, stress	1 (0–3)	0 (0–3)	1 (0–5)	2.871	0.004	0 (0–3)	1 (0–5)	3.350	0.001	0 (0–3)	1 (0–4)	2.102	0.036
PSQI, insomnia	3 (2–5)	3 (2–5)	3 (2–6)	0.553	0.581	3 (2–5)	3 (2–6)	2.050	0.040	3 (2–5)	3 (2–6)	1.402	0.161

*PTSD, post-traumatic stress disorder; IES-R, impact of event scale–revised; DASS, depression, anxiety, and stress scale; PSQI, Pittsburgh sleep quality index*.

**Table 3:** Severity Classifications of PTSD, depression, anxiety, stress, and insomnia symptoms.

	**Total**	**Educational status**				**Gender**				**Location**			
**Variables**	***N*** **= 1,532**	**Junior** ***N*** **= 1,135**	**Senior** ***N*** **= 397**	***Z***	***P***	**Female** ***N*** **= 1,351**	**Male** ***N*** **= 181**	***Z***	***P***	**Rural** ***N*** **= 866**	**Urban** ***N*** **= 666**	***Z***	***P***
**IES-R, PTSD**				3.318	0.001			2.994	0.003			3.122	0.002
Normal	850 (55.5)	655 (57.7)	195 (49.1)			768 (56.8)	82 (45.3)			510 (58.9)	340 (51.1)		
Mild	636 (41.5)	455 (40.1)	181 (45.6)			545 (40.3)	91 (50.3)			334 (38.6)	302 (45.3)		
Moderate	41 (2.7)	24 (2.1)	17 (4.3)			34 (2.5)	7 (3.9)			21 (2.4)	20 (3.0)		
Severe	5 (0.3)	1 (0.1)	4 (1.0)			4 (0.3)	1 (0.6)			1 (0.1)	4 (0.6)		
**DASS, depression**				3.561	<0.001			2.651	0.008			2.577	0.010
Normal	1,487 (97.1)	1,112 (98.0)	375 (94.5)			1,317 (97.5)	170 (93.9)			849 (98.0)	638 (95.8)		
Mild	24 (1.6)	11 (1.0)	13 (3.3)			17 (1.3)	7 (3.9)			9 (1.0)	15 (2.3)		
Moderate	19 (1.2)	12 (1.1)	7 (1.8)			16 (1.2)	3 (1.7)			8 (0.9)	11 (1.7)		
Severe	2 (0.1)	0 (0.0)	2 (0.5)			1 (0.1)	1 (0.6)			0 (0.0)	2 (0.3)		
**DASS, anxiety**				3.719	<0.001			3.224	0.001			2.448	0.014
Normal	1,488 (97.1)	1,113 (98.1)	375 (94.5)			1,319 (97.6)	169 (93.4)			849 (98.0)	639 (95.9)		
Mild	26 (1.7)	15 (1.3)	11 (2.8)			19 (1.4)	7 (3.9)			12 (1.4)	14 (2.1)		
Moderate	15 (1.0)	6 (0.5)	9 (2.3)			11 (0.8)	4 (2.2)			5 (0.6)	10 (1.5)		
Severe	3 (0.2)	1 (0.1)	2 (0.5)			2 (0.1)	1 (0.6)			0 (0.0)	3 (0.5)		
**DASS, stress**				2.007	0.045			2.268	0.023			2.274	0.023
Normal	1,515 (98.9)	1,126 (99.2)	389 (98.0)			1,339 (99.1)	176 (97.2)			861 (99.4)	654 (98.2)		
Mild	13 (0.8)	8 (0.7)	5 (1.3)			10 (0.7)	3 (1.7)			5 (0.6)	8 (1.2)		
Moderate	3 (0.2)	1 (0.1)	2 (0.5)			2 (0.1)	1 (0.6)			0 (0.0)	3 (0.5)		
Severe	1 (0.1)	0 (0.0)	1 (0.3)			0 (0.0)	1 (0.6)			0 (0.0)	1 (0.2)		
**PSQI, insomnia**				1.222	0.222			2.614	0.009			1.482	0.138
Normal	1,160 (75.7)	868 (76.5)	292 (73.6)			1,036 (76.7)	124 (68.5)			668 (77.1)	492 (73.9)		
Mild	332 (21.7)	240 (21.1)	92 (23.2)			286 (21.2)	46 (25.4)			177 (20.4)	155 (23.3)		
Moderate	38 (2.5)	26 (2.3)	12 (3.0)			27 (2.0)	11 (6.1)			21 (2.4)	17 (2.6)		
Severe	2 (0.1)	1 (0.1)	1 (0.3)			2 (0.1)	0 (0.0)			0 (0.0)	2 (0.3)		

*PTSD, post-traumatic stress disorder; IES-R, impact of event scale–revised; DASS, depression, anxiety, and stress scale; PSQI, Pittsburgh sleep quality index*.

Moreover, the results of subgroup analysis indicated that as compared with juniors and female nursing students, senior interns and male nursing students reported higher scores of PTSD [senior 9 (3–15) vs. junior 7 (3–14), *P* < 0.01; male 11 (3–16) vs. female 7 (3–14), *P* < 0.01], depression [senior 1 (0–4) vs. junior 0 (0–2), *P* < 0.01; male 1 (0–5) vs. female 0 (0–2), *P* < 0.01], anxiety [senior 0 (0–3) vs. junior 0 (0–1), *P* < 0.05; male 0 (0–3) vs. female 0 (0–2), *P* < 0.01], and stress [senior 1 (0–5) vs. junior 0 (0–3), *P* < 0.01; male 1 (0–5) vs. female 0 (0–3), *P* < 0.01]. As compared with rural nursing students, urban nursing students also reported higher scores of PTSD [8 (3–15) vs. 7 (3–13), *P* < 0.05], anxiety [0 (0–2) vs. 0 (0–1), *P* < 0.05], stress [1 (0–4) vs. 0 (0–3), *P* < 0.05], and depression [0 (0–3) vs. rural 0 (0–2), *P* = 0.072]. Accordingly, as compared with junior, female, and rural nursing students, these senior, male, and urban nursing students had higher incidences of symptoms of PTSD (senior 50.9% vs. junior 42.3%, *P* < 0.01; male 54.7% vs. female 43.2%, *P* < 0.01; urban 48.9% vs. rural 41.1%, *P* < 0.01), depression (senior 5.5% vs. junior 2.0%, *P* < 0.01; male 6.1% vs. female 2.5%, *P* < 0.01; urban 4.2% vs. rural 2.0%, *P* < 0.05), anxiety (senior 5.5% vs. junior 1.9%, *P* < 0.01; male 6.6% vs. female 2.4%, *P* < 0.01; urban 4.1% vs. rural 2.0%, *P* < 0.05), and stress (senior 2.0% vs. junior 0.8%, *P* < 0.05; male 2.8% vs. female 0.9%, *P* < 0.05; urban 1.8% vs. rural 0.6%, *P* < 0.05). Furthermore, male nursing students had higher PSQI scores [3 (2–6) vs. 3 (2–5), *P* < 0.05] and rates of insomnia (31.5 vs. 23.3%, *P* < 0.05) when compared with female nursing students. The detailed mental health outcomes are also shown in Tables 2, 3.

### Risk Factors for Mental Health of Nursing Students

The results of multivariable logistic regression analysis are presented in Table 4. As compared with junior nursing students, senior nursing students (interns) had a higher risk of PTSD (OR = 1.406; 95% CI, 1.116–1.771; *P* = 0.004), depression (OR = 2.790; 95% CI, 1.529–5.093; *P* = 0.001), and anxiety (OR = 2.950; 95% CI, 1.604–5.425; *P* < 0.001). As compared with female nursing students, male nursing students had higher risks of PTSD (OR = 1.631; 95% CI, 1.192–2.233; *P* = 0.002), depression (OR = 2.676; 95% CI, 1.318–5.431; *P* = 0.006), anxiety (OR = 3.144; 95% CI, 1.572–6.286; *P* = 0.001), stress (OR = 3.352; 95% CI, 1.158–9.702; *P* = 0.026), and insomnia (OR = 1.478; 95% CI, 1.048–2.084; *P* = 0.026). As compared with rural nursing students, urban nursing students had higher risks of PTSD (OR = 1.362; 95% CI, 1.110–1.672; *P* = 0.003), depression (OR = 2.091; 95% CI, 1.129–3.874; *P* = 0.019), anxiety (OR = 2.010; 95% CI, 1.079–3.743; *P* = 0.028), and stress (OR = 3.031; 95% CI, 1.057–8.693; *P* = 0.039).

**Table 4:** Logistic regression analysis for PTSD, depression, anxiety, stress, and insomnia risk factors.

**Variables**	**Abnormal/total** **cases (%)**	**Goodness** **of fit**	**B**	**SE**	**Wald**	***P***	**OR**	**95% CI**
**IES-R, PTSD**	682/1,532 (44.5)	0.916						
**Educational status**								
Junior [reference]	480/1,135 (42.3)		–	–	–	–	1	–
Senior interns	202/397 (50.9)		0.341	0.118	8.352	0.004	1.406	1.116–1.771
**Gender**								
Female [reference]	583/1,351 (43.2)		–	–	–	–	1	–
Male	99/181 (54.7)		0.489	0.160	9.333	0.002	1.631	1.192–2.233
**Location**								
Rural [reference]	356/866 (41.1)		–	–	–	–	1	–
Urban	326/666 (48.9)		0.309	0.105	8.743	0.003	1.362	1.110–1.672
**DASS, depression**	45/1,532 (2.9)	0.069						
**Educational status**								
Junior [reference]	23/1,135 (2.0)		–	–	–	–	1	–
Senior (interns)	22/397 (5.5)		1.026	0.307	11.176	0.001	2.790	1.529–5.093
**Gender**								
Female [reference]	34/1,351 (2.5)		–	–	–	–	1	–
Male	11/181 (6.1)		0.984	0.361	7.424	0.006	2.676	1.318–5.431
**Location**								
Rural [reference]	17/866 (2.0)		–	–	–	–	1	–
Urban	28/666 (4.2)		0.738	0.315	5.498	0.019	2.091	1.129–3.874
**DASS, anxiety**	44/1,532 (2.9)	0.893						
**Educational status**								
Junior [reference]	22/1,135 (1.9)		–	–	–	–	1	–
Senior (interns)	22/397 (5.5)		1.082	0.311	12.116	<0.001	2.950	1.604–5.425
**Gender**								
Female [reference]	32/1,351 (2.4)		–	–	–	–	1	–
Male	12/181 (6.6)		1.145	0.354	10.498	0.001	3.144	1.572–6.286
**Location**								
Rural [reference]	17/866 (2.0)		–	–	–	–	1	–
Urban	27/666 (4.1)		0.698	0.317	4.837	0.028	2.010	1.079–3.743
**DASS, stress**	17/1,532 (1.1)	0.091						
**Educational status**								
Junior [reference]	9/1,135 (0.8)		–	–	–	–	1	–
Senior (interns)	8/397 (2.0)		0.904	0.493	3.355	0.067	2.468	0.939–6.490
**Gender**								
Female [reference]	12/1,351 (0.9)		–	–	–	–	1	–
Male	5/181 (2.8)		1.209	0.542	4.974	0.026	3.352	1.158–9.702
**Location**								
Rural [reference]	5/866 (0.6)		–	–	–	–	1	–
Urban	12/666 (1.8)		1.109	0.538	4.256	0.039	3.031	1.057–8.693
**PSQI, insomnia**	358/1,532 (23.4)	0.731						
**Educational status**								
Junior [reference]	259/1,135 (22.8)		–	–	–	–	1	–
Senior (interns)	99/397 (24.9)		0.114	0.136	0.695	0.404	1.120	0.858–1.464
**Gender**								
Female [reference]	304/1,351 (22.5)		–	–	–	–	1	–
Male	54/181 (29.8)		0.391	0.175	4.962	0.026	1.478	1.048–2.084
**Location**								
Rural [reference]	190/866 (21.9)		–	–	–	–	1	–
Urban	168/666 (25.2)		0.182	0.122	2.225	0.136	1.199	0.945–1.522

*PTSD, post-traumatic stress disorder; IES-R, impact of event scale–revised; DASS, depression, anxiety, and stress scale; PSQI, Pittsburgh sleep quality index*.

## Discussion

In this study, we conducted a survey to evaluate the mental health condition of college nursing students amid the COVID-19 pandemic. Measurement scales including IES-R, DASS-21, and PSQI were used to assess the symptoms of PTSD, depression, anxiety and stress, and insomnia, respectively. We found that among the college nursing students, 44.5% presented with symptoms of PTSD, 2.9% with depression, 2.9% with anxiety, 1.1% with stress, and 22.8% presented with symptoms of insomnia. Fortunately, few nursing students experienced severe symptoms of PTSD (0.3%), depression (0.1%), anxiety (0.2%), stress (0.1%), and insomnia (0.1%). Additionally, as compared with junior, female, and rural nursing students, senior (interns), male, and urban nursing students had higher occurrence rates of PTSD, depression, anxiety, and stress, and male nursing students had a higher rate of insomnia. Surprisingly, the results showed that an overwhelming majority of students had a positive attitude (98.2%) about becoming frontline nurses against COVID-19 despite the high risk of contagion.

At present, there is still no definitive treatment for COVID-19, we do not know how long this will last, and the future remains unpredictable. The psychological impact of the COVID-19 pandemic is extensive and profound, as it has led to psychological symptoms such as fear, irritability, uncertainty, PTSD, depression, anxiety, stress, and insomnia among people all over the world. Lockdowns, economic losses, and the lack of masks and alcohol-based disinfectants further aggravated feelings of social isolation, loneliness, and the above negative psychological symptoms and even gave rise to delirium, self-harm, and suicide (Ettman et al., 2020; Twenge and Joiner, 2020). A large-scale online survey showed that the rates of mental health symptoms among the general Chinese population from February 28 to March 11, 2020 showed that 27.9% had symptoms of depression, 31.6% had symptoms of anxiety, 29.2% had symptoms of insomnia, and 24.4% experienced acute stress (Shi et al., 2020). Another large web-based survey conducted in the United States from June 24 to 30, 2020 indicated that 26.3, 24.3, and 25.5% of the adult respondents presented with symptoms of PTSD, depression, and anxiety, respectively, and 10.7% respondents (8.9% females and 12.6% males) had seriously considered suicide in the previous 30 days (Czeisler et al., 2020).

Previous studies have indicated that healthcare workers had high levels of stress, depression, and anxiety. Furthermore, healthcare workers, especially nurses who were directly exposed to COVID-19 due to circumstances such as serving in isolation wards or emergency departments had higher levels of adverse psychiatric outcomes (Azoulay et al., 2020; Lai et al., 2020; Si et al., 2020). For instance, a survey regarding the mental health of healthcare workers in China revealed that the rates of PTSD, depression, anxiety, and insomnia were 71.5, 50.4, 44.6, and 34.0%, respectively, among all the participants, whereas these values were 74.5, 53.5, 47.1, and 38.2% among nurses, respectively (Lai et al., 2020). These findings were more severe than the results of this study.

Some recent studies revealed that students also experienced adverse mental symptoms after the COVID-19 outbreak, and the mental effects of COVID-19 may differ among countries and areas due to discrepancies in the COVID-19 infection and anti-epidemic conditions. First, a cross-sectional study regarding the psychological effects of the COVID-19 outbreak and lockdown among students (76.8%) and workers (23.2%) in a university in Spain which was severely affected by the COVID-19 pandemic showed that 87.5, 48.1, 35.2, and 40.3% of the respondents presented with symptoms of PTSD, depression, anxiety, and stress, respectively, with students having higher scores of depression, anxiety, and stress (Odriozola-Gonzalez et al., 2020). Next, a survey conducted in Texas A&M University, USA which was also severely affected by the COVID-19 pandemic showed that 80.6 and 71.8% of respondents reported symptoms of depression and anxiety, respectively (Wang X. et al., 2020). Furthermore, a survey about the impact of the COVID-19 pandemic on the mental health of home-quarantined students in Bangladesh showed that 69.3, 46.9, 33.3, and 28.5% of respondents reported having symptoms of PTSD, depression, anxiety, and stress, respectively (Khan et al., 2020). Finally, a large cross-sectional survey conducted among college students in Guangdong Province, China which aimed to assess the psychological impact of the COVID-19 outbreak showed that 50.9% respondents had abnormal IES scores, 0.5% reported poor mental health, and 3.2% reported poor sleep quality (Li X. et al., 2020).

As for nursing students, a recently published survey conducted from March 8 to 24, 2020 in China showed that the prevalence of anxiety, depression, and PTSD were 34.97, 40.22, and 14.97%, respectively (Li et al., 2021). Additionally, a multicenter cross-sectional study conducted from April 30 to May 14, 2020 in three European countries (Spain, Greece, and Albania) indicated that 67.5% nursing students experienced mild to severe depression, and the rates of depression differed among countries (Spain, 86%; Greece, 59.5% and Albania, 58.9%) (Patelarou et al., 2021). In this study, although the incidence rates of anxiety and depression were lower than that of the above studies, about half (44.5%) of the respondents reported symptoms of PTSD, whereas about a quarter (22.8%) reported symptoms of insomnia. Therefore, the psychological impact of the COVID-19 pandemic on nursing students is considerable, and special psychological guidance, support, and interventions should be implemented to assure their mental health.

Based on the above studies, we could conclude that the incidences of mental symptoms might be greater in areas with a high risk of COVID-19 or during high-risk periods. However, these results may be influenced by the use of different scales to evaluate the same symptoms. For example, depression was assessed using the Patient Health Questionnaire 9 (PHQ-9) in the above two studies; however, we used the DASS-21 to assess depression in this study.

In this study, senior and urban nursing students indicated higher levels of mental symptoms. Senior nursing students (interns) had started working under their clinical practice which required them to come into contact with all kinds of patients. As they would become clinical staff nurses soon, their feelings about the COVID-19 contagion might be more intuitive and deeper than junior nursing students which may have caused them to experience more symptoms of PTSD, depression, anxiety, and stress, even though all of them were required by the education department to study at home. In addition, their worries regarding their clinical skills and further education or employment may contribute to their higher levels of mental symptoms. Due to the dense population and convenient transportation in urban areas (about 2 h from Wuhan city to Zhengzhou city by high-speed train), the spread of COVID-19 was more severe in urban areas compared with rural areas, which may have resulted in the higher incidence of mental symptoms among urban nursing students.

Unexpectedly, compared with female nursing students, male nursing students reported higher incidences of symptoms of PTSD, depression, anxiety, and stress in this study, which was different from previous studies about healthcare workers (Lai et al., 2020). Possible reasons for this inconsistency are listed as follows. First, the respondents in this study were far from the areas with a high-risk of COVID-19 and were relatively safe at home. Second, male nursing students paid more attention to the COVID-19 outbreak (always, 50.8 vs. 40.0% among females). Finally, male nursing students had higher rates of insomnia (31.5 vs. 23.3%).

As more severe mental symptoms of PTSD, depression, anxiety, and stress were found in senior, urban, and male nursing students, governments, schools, and teachers should pay more attention to students with these risk factors. More frontline or online mental health counseling and support should be provided for these students to promote their mental health. First, senior nursing students should be educated more regarding COVID-19 prevention and treatment, and governments and schools should guarantee their chances of clinical practice and employment. Second, as for junior nursing students, it is important to educate them regarding COVID-19, and strengthening their professional identity, ideals, and faith may help prevent them from experiencing more severe psychological symptoms. Third, there are fewer male nursing students in China, and even though it is easier for them to get jobs compared with female nursing students, they had poor professional feelings of self-identity, responsibility, honor, and pride; thus, they may require special attention and more relevant education. Furthermore, nursing students from urban cities which had a higher risk of COVID-19 infection require more education regarding the prevention and control of COVID-19 infection, such as maintaining social distancing, wearing masks, and hand hygiene, and the government should provide them with timely, updated, and accurate official information regarding COVID-19.

## Conclusion

In this study, a considerable number of nursing students reported having symptoms of PTSD and insomnia, whereas few nursing students reported mental symptoms of depression, anxiety, and stress. Furthermore, senior, male, and urban nursing students may be at risk for more severe mental symptoms. As nursing students are an important reserve force against the COVID-19 pandemic, special psychological interventions should be implemented to assure their mental health.

## Limitations

There were several limitations in our study. First, this study had an online cross-sectional design. Second, we only investigated the nursing students from one college in Henan Province, China, so the findings may differ among other colleges, areas, or populations such as students belonging to other disciplines. Furthermore, as fewer respondents reported symptoms of depression, anxiety, and stress, the sample may have been insufficient for the subgroup analysis. Finally, we did not assess the effects of psychiatric and physical disorders of respondents, which might affect the results. Based on the above limitations, further studies regarding the mental health of nursing students should be conducted in the future.

## Data Availability Statement

The raw data supporting the conclusions of this article will be made available by the authors, without undue reservation.

## Author Contributions

FH, JG, and FW conceived and designed the experiments. JG wrote the original draft. JG, FW, and SG performed data collection, analysis, and interpretation. FH reviewed and edited the manuscript. All authors contributed to this study and approved the final version of the manuscript.

## Conflict of Interest

The authors declare that the research was conducted in the absence of any commercial or financial relationships that could be construed as a potential conflict of interest.

## Publisher's Note

All claims expressed in this article are solely those of the authors and do not necessarily represent those of their affiliated organizations, or those of the publisher, the editors and the reviewers. Any product that may be evaluated in this article, or claim that may be made by its manufacturer, is not guaranteed or endorsed by the publisher.
