# Supplementary material for: Mental Health of Nursing Students amid Coronavirus Disease 2019 Pandemic
Source: Front Psychol. 2021 Aug 12;12:699558. doi: 10.3389/fpsyg.2021.699558 (PMC8407077; doi:10.3389/fpsyg.2021.699558)
Supplement: Supplementary file 1 [file Data_Sheet_1.PDF]

Supplementary Material

Investigation on Mental Health Status of Nursing College Students During COVID-19 Pandemic

Dear students,

The COVID-19 epidemic is sweeping the world and touching the heart of every one of us. Nowadays, we are all working together to overcome the epidemic! In order to understand the mental health status of nursing college students during the epidemic, we conduct this survey now. We sincerely hope you can spare your precious time to fill in this questionnaire seriously and truthfully. This questionnaire is anonymous, and your information will be strictly confidential. The survey results are only for scientific research, please feel free to fill in.

Thank you very much for your support and participation!

Please read each statement and select the suitable items applied to you. Please try to answer every question.

1. What is your grade? [single choice] *

○ Freshman (Grade 1)

○ Sophomore (Grade 2)

○ Senior (Grade 3)

2. What is your major in college? [single choice] *

○ Clinical medicine

○ Nursing

○ Midwifery
○ Other_________

3. What is your gender? [single choice] *

○ Male ○ female

4. What is your birth date (e.g. 200101) ? [fill in the blank] *

____

5. Your blood type: [single choice] *

○ A

○ B

○ AB

○ O

○ Unclear

6. Please select your province, city and region: [fill in the blank] *

____

Impact of COVID-19 Event Scale-Revised (IES-R)

7. Any reminder bring back feelings about COVID-19 experience. [**IES-R 1**, single choice] *

**Items**	Not at all	A little bit	Moderately	Quite a bit	Extremely
**Options**	○	○	○	○	○

8. I have trouble staying asleep. [**IES-R 2**, single choice] *

**Items**	Not at all	A little bit	Moderately	Quite a bit	Extremely
**Options**	○	○	○	○	○

9. Other things keep making me think about COVID-19. [**IES-R 3**, single choice] *

Items	Not at all	A little bit	Moderately	Quite a bit	Extremely
**Options**	○	○	○	○	○

10. I feel irritable and angry easily. [**IES-R 4**, single choice] *

Items	Not at all	A little bit	Moderately	Quite a bit	Extremely
**Options**	○	○	○	○	○

11. When I think about or remind of COVID-19, I try not to make myself sad. [**IES-R 5**, single choice] *

Items	Not at all	A little bit	Moderately	Quite a bit	Extremely
**Options**	○	○	○	○	○

12. I don't want to, but I think about COVID-19. [**IES-R 6**, single choice] *

Items	Not at all	A little bit	Moderately	Quite a bit	Extremely
**Options**	○	○	○	○	○

13. I feel as if COVID-19 didn't happen or wasn't real. [**IES-R 7**, single choice] *

Items	Not at all	A little bit	Moderately	Quite a bit	Extremely
**Options**	○	○	○	○	○

14. I try to stay away from reminders about COVID-19. [**IES-R 8**, single choice] *

Items	Not at all	A little bit	Moderately	Quite a bit	Extremely
**Options**	○	○	○	○	○

15. Pictures about COVID-19 often pop into my mind. [**IES-R 9**, single choice] *

Items	Not at all	A little bit	Moderately	Quite a bit	Extremely
**Options**	○	○	○	○	○

16. I feel jumpy and easily startled. [**IES-R 10**, single choice] *

Items	Not at all	A little bit	Moderately	Quite a bit	Extremely
Options	○	○	○	○	○

17. I try not to think about COVID-19. [**IES-R 11**, single choice] *

Items	Not at all	A little bit	Moderately	Quite a bit	Extremely
Options	○	○	○	○	○

18. I am aware that I still have a lot of feelings about COVID-19, but I don't deal with them. [**IES-R 12**, single choice] *

Items	Not at all	A little bit	Moderately	Quite a bit	Extremely
Options	○	○	○	○	○

19. My feelings about COVID-19 are kind of numb. [**IES-R 13**, single choice] *

Items	Not at all	A little bit	Moderately	Quite a bit	Extremely
Options	○	○	○	○	○

20. I find myself acting or feeling as though I am back at the time of COVID-19 pandemic started. [**IES-R 14**, single choice] *

Items	Not at all	A little bit	Moderately	Quite a bit	Extremely
Options	○	○	○	○	○

21. I have trouble falling asleep. [**IES-R 15**, single choice] *

Items	Not at all	A little bit	Moderately	Quite a bit	Extremely
Options	○	○	○	○	○

22. I still often have waves of strong feelings about COVID-19. [**IES-R 16**, single choice]*

Items	Not at all	A little bit	Moderately	Quite a bit	Extremely
Options	○	○	○	○	○

23. I have tried to remove COVID-19 from my memory. [**IES-R 17**, single choice] *

**Items**	Not at all	A little bit	Moderately	Quite a bit	Extremely
**Options**	○	○	○	○	○

24. I have trouble concentrating. [**IES-R 18**, single choice] *

**Items**	Not at all	A little bit	Moderately	Quite a bit	Extremely
**Options**	○	○	○	○	○

25. Reminders of COVID-19 caused me to have physical reactions, such as sweating, trouble breathing, nausea, or a pounding heart. [**IES-R 19**, single choice] *

**Items**	Not at all	A little bit	Moderately	Quite a bit	Extremely
**Options**	○	○	○	○	○

26. I have dreams related to COVID-19. [**IES-R 20**, single choice] *

**Items**	Not at all	A little bit	Moderately	Quite a bit	Extremely
**Options**	○	○	○	○	○

27. I feel watchful or on-guard. [**IES-R 21**, single choice] *

**Items**	Not at all	A little bit	Moderately	Quite a bit	Extremely
**Options**	○	○	○	○	○

28. I try not to talk about COVID-19. [**IES-R 22**, single choice] *

**Items**	Not at all	A little bit	Moderately	Quite a bit	Extremely
**Options**	○	○	○	○	○

29. I think the COVID-19 epidemic have influenced me. [additional single choice] *

**Items**	Greatly	Quite a bit	Moderately	A little bit	Not at all
**Options**	○	○	○	○	○

30. During the domestic epidemic, I feel my life was greatest influenced by COVID-19 epidemic… [additional single choice] *

**Items**	At the beginning	At the peak	At current stable period
**Options**	○	○	○

31. During the domestic epidemic, I feel my life was least influenced by COVID-19 epidemic… [additional single choice] *

**Items**	At the beginning	At the peak	At current stable period
**Options**	○	○	○

Depression, Anxiety and Stress Scale (DASS)-21 Emotional Self-Rating

32. I find it difficult to wind down. [**DASS 1**, single choice] *

○ Do not apply to me at all

○ Apply to me to some degree or some of the time

○ Apply to me a considerable degree or a good part of the time

○ Apply to me very much or most of the time

33. I am aware of dryness of my mouth. [**DASS 2**, single choice] *

○ Do not apply to me at all

○ Apply to me to some degree or some of the time

○ Apply to me a considerable degree or a good part of the time

○ Apply to me very much or most of the time

34. I can’t seem to experience any positive feeling at all. [**DASS 3**, single choice] *

○ Do not apply to me at all

○ Apply to me to some degree or some of the time

○ Apply to me a considerable degree or a good part of the time

○ Apply to me very much or most of the time

35. I have experienced breathing difficulty (eg, excessively rapid breathing, breathlessness in the absence of physical exertion). [**DASS 4**, single choice] *

○ Do not apply to me at all

○ Apply to me to some degree or some of the time

○ Apply to me a considerable degree or a good part of the time

○ Apply to me very much or most of the time

36. I find it difficult to work up the initiative to do things. [**DASS 5**, single choice] *

○ Not at all

○ Partial conformity

○ Most of them agree

○ It fits very well

37. I tend to over-react to situations. [**DASS 6**, single choice] *

○ Do not apply to me at all

○ Apply to me to some degree or some of the time

○ Apply to me a considerable degree or a good part of the time

○ Apply to me very much or most of the time

38. I have experienced trembling (eg, in the hands). [**DASS 7**, single choice] *

○ Do not apply to me at all

○ Apply to me to some degree or some of the time

○ Apply to me a considerable degree or a good part of the time

○ Apply to me very much or most of the time

39. I feel that I am using a lot of nervous energy. [**DASS 8**, single choice] *

○ Do not apply to me at all

○ Apply to me to some degree or some of the time

○ Apply to me a considerable degree or a good part of the time

○ Apply to me very much or most of the time

40. I am worried about situations in which I might panic and make a fool of myself. [**DASS 9**, single choice] *

○ Do not apply to me at all

○ Apply to me to some degree or some of the time

○ Apply to me a considerable degree or a good part of the time

○ Apply to me very much or most of the time

41. I feel that I had nothing to look forward to. [**DASS 10**, single choice] *

○ Do not apply to me at all

○ Apply to me to some degree or some of the time

○ Apply to me a considerable degree or a good part of the time

○ Apply to me very much or most of the time

42. I find myself getting agitated. [**DASS 11**, single choice] *

○ Do not apply to me at all

○ Apply to me to some degree or some of the time

○ Apply to me a considerable degree or a good part of the time

○ Apply to me very much or most of the time

43. I find it difficult to relax. [**DASS 12**, single choice] *

○ Do not apply to me at all

○ Apply to me to some degree or some of the time

○ Apply to me a considerable degree or a good part of the time

○ Apply to me very much or most of the time

44. I feel down-hearted and blue. [**DASS 13**, single choice] *

○ Do not apply to me at all

○ Apply to me to some degree or some of the time

○ Apply to me a considerable degree or a good part of the time

○ Apply to me very much or most of the time

45. I am intolerant of anything that keep me from getting on with what I am doing. [**DASS 14**, single choice] *

○ Do not apply to me at all

○ Apply to me to some degree or some of the time

○ Apply to me a considerable degree or a good part of the time

○ Apply to me very much or most of the time

46. I feel I am close to panic. [**DASS 15**, single choice] *

○ Do not apply to me at all

○ Apply to me to some degree or some of the time

○ Apply to me a considerable degree or a good part of the time

○ Apply to me very much or most of the time

47. I am unable to become enthusiastic about anything. [**DASS 16**, single choice] *

○ Do not apply to me at all

○ Apply to me to some degree or some of the time

○ Apply to me a considerable degree or a good part of the time

○ Apply to me very much or most of the time

48. I feel I am not worth much as a person. [**DASS 17**, single choice] *

○ Do not apply to me at all

○ Apply to me to some degree or some of the time

○ Apply to me a considerable degree or a good part of the time

○ Apply to me very much or most of the time

49. I feel that I am rather touchy. [**DASS 18**, single choice] *

○ Do not apply to me at all

○ Apply to me to some degree or some of the time

○ Apply to me a considerable degree or a good part of the time

○ Apply to me very much or most of the time

50. I am aware of the action of my heart in the absence of physical exertion (eg, sense of heart rate increase, heart missing a beat). [**DASS 19**, single choice] *

○ Do not apply to me at all

○ Apply to me to some degree or some of the time

○ Apply to me a considerable degree or a good part of the time

○ Apply to me very much or most of the time

51. I feel scared without any good reason. [**DASS 20**, single choice]*

○ Do not apply to me at all

○ Apply to me to some degree or some of the time

○ Apply to me a considerable degree or a good part of the time

○ Apply to me very much or most of the time

52. I feel that life has no meaning. [**DASS 21**, single choice] *

○ Do not apply to me at all

○ Apply to me to some degree or some of the time

○ Apply to me a considerable degree or a good part of the time

○ Apply to me very much or most of the time

53. My emotional and psychological stress during the epidemic... [additional single choice] *

○ significantly increase

○ increase

○ have no change

○ decrease

○ significantly decrease

54. During the domestic epidemic, I feel the most severe emotional and psychological stress… [additional single choice] *

○ at the beginning

○ at the peak

○ at current stable period

55. During the domestic epidemic, I feel the least emotional and psychological stress… [additional single choice] *

○ at the beginning

○ at the peak

○ at current stable period

56. What are the causes of your stress during the COVID-19 epidemic? [additional multiple choice] *

☐ Physical health

☐ Lack of recreational activities

☐ Less economic income

☐ Studies are affected

☐ Poor diet

☐ Poor sleep

☐ No stress at all

57. If you have bad feelings, they mainly come from the following aspects. [additional multiple choice] *

☐ Lack of knowledge about COVID-19 disease

☐ Protective equipment is not available

☐ Unable to travel normally

☐ Worried about being infected

☐ Worried about not being able to see a doctor

☐ Worried about family members getting infected

☐ See that the epidemic data is increasing every day

☐ Unable to study normally

☐ other

58. When you have bad emotions, how do you deal with? [additional multiple choice] *

☐ Listen to music

☐ Play the game

☐ Talk with friends

☐ Self-elimination

☐ Play sports

☐ Others

Pittsburgh Sleep Quality Index (PSQI) Scale

59. During the past month, what time have you usually gone to bed at night? ______ (**PSQI 1**, 24-hour time, blank filling) *

60. During the past month, how long (in minutes) has it usually taken you to fall asleep each night? [**PSQI 2**, single choice] *

○ Within 15 minutes

○ 16 to 30 minutes

○ 31 to 60 minutes

○ More than 60 minutes

61. During the past month, I usually get up at_____ (24-hour time) in the morning. [**PSQI 3**, blank filling]*

62. During the past month, I usually sleep_____ hours (not equal to your bed time) every night [**PSQI 4**, blank filling]*

How often have you had trouble sleeping, because you…[**PSQI 5**]*

63. Cannot get to sleep within 30 min during the past month. [**PSQI 5a**, single choice] *

○ Not during the past month

○ Less than once a week

○ Once or twice a week

○ Three or more times week

64. Wake up in the middle of the night or early morning during the past month. [**PSQI 5b**, single choice] *

○ Not during the past month

○ Less than once a week

○ Once or twice a week

○ Three or more times week

65. Have to get up to use the bathroom during the past month. [**PSQI 5c**, single choice] *

○ Not during the past month

○ Less than once a week

○ Once or twice a week

○ Three or more times week

66. Cannot breathe comfortably during the past month. [**PSQI 5d**, single choice] *

○ Not during the past month

○ Less than once a week

○ Once or twice a week

○ Three or more times week

67.Cough or snore loudly during the past month. [**PSQI 5e**, single choice] *

○ Not during the past month

○ Less than once a week

○ Once or twice a week

○ Three or more times week

68. Feel too cold during the past month. [**PSQI 5f**, single choice] *

○ Not during the past month

○ Less than once a week

○ Once or twice a week

○ Three or more times week

69. Feel too hot during the past month. [**PSQI 5g**, single choice] *

○ Not during the past month

○ Less than once a week

○ Once or twice a week

○ Three or more times week

70. Had bad dreams during the past month. [**PSQI 5h**, single choice] *

○ Not during the past month

○ Less than once a week

○ Once or twice a week

○ Three or more times week

71. Have pain during the past month. [**PSQI 5i**, single choice] *

○ Not during the past month

○ Less than once a week

○ Once or twice a week

○ Three or more times week

72. Have other reason(s) affecting the sleep during the past month. [**PSQI 5j**, single choice] *

○ Not during the past month

○ Less than once a week

○ Once or twice a week

○ Three or more times week

73. During the past month, how would you rate your sleep quality overall? [**PSQI 6**, single choice] *

○ Very good

○ Fairly good

○ Fairly bad

○ Very bad

74. During the past month, how often have you taken medicine (prescribed or "over the counter") to help you sleep? [**PSQI 7**, single choice] *

○ Not during the past month

○ Less than once a week

○ Once or twice a week

○ Three or more times week

75. During the past month, how often have you bad trouble staying awake while driving, eating meals, or engaging in social activity? [**PSQI 8**, single choice] *

○ Not during the past month

○ Less than once a week

○ Once or twice a week

○ Three or more times week

76. During the past month, how much of a problem has it been for you to keep up enough enthusiasm to get things done? [**PSQI 9**, single choice] *

○ No problem at all

○ Only a very slight problem

○ Somewhat of a problem

○ A very big problem

77. How would you rate your sleep quality during the COVID-19 epidemic. [additional single choice] *

○ Obviously better

○ Better

○ No changes

○ Worse

○ Obviously worse

78. When do you get the best sleep during the domestic COVID-19 epidemic. [additional single choice] *

○ At the beginning

○ At the peak

○ At current stable period

79. When do you get the worst sleep quality during the domestic COVID-19 epidemic. [additional single choice] *

○ At the beginning

○ At the peak

○ At current stable period

Social Support Rating Scale (SSRS)

80. Are you an only child?   [single choice] *

○ Yes

○ No

81. Where are you located in? [single choice] *

○ Town

○ The rural

82. How many close friends do you have whom you can rely on for support and help? [single choice] *

○ No one

○ 1-2

○ 3-5

○ 6 or more

83. In the past year, you…[single choice] *

○ lived in a single room away from family

○ changed residence frequently and lived with strangers most of the time

○ lived with classmates or friends

○ lived with family

84. What is the relationship between you and your neighbors? [single choice] *

○ Never care about each other, just bowing acquaintances

○ They can care you a little when you encounter difficulties

○ Some of your neighbors or classmates care a lot about you

○ Most of the neighbors or classmates care a lot about you

85. What is the relationship between you and your classmates? [single choice] *

○ Never care about each other, just bowing acquaintances

○ They can care you a little when you encounter difficulties

○ Some of your neighbors or classmates care a lot about you

○ Most of the neighbors or classmates care a lot about you

86. How about support and care received from your family members? [single choice] *

	No	Very few	Generally	Fully
Lover	○	○	○	○
Parents	○	○	○	○
Brothers and sisters	○	○	○	○
Other (e.g. uncle)	○	○	○	○

87. In the past, when you encountered an emergency, you had received financial support and practical help from the following sources: [single or multiple choice] *

☐ without any source

☐ lover

☐ family

☐ relatives

☐ classmates

☐ school

☐ party committee, associations and other official or semi-official organizations

☐ non-official organizations such as religious and social groups

☐ other

88. In the past, when you encountered an emergency, you had been comforted and cared for by the following sources: [single or multiple choice] *

☐ without any source

☐ lover

☐ family

☐ relatives

☐ classmates

☐ school

☐ party committee, associations and other official or semi-official organizations

☐ non-official organizations such as religious and social groups

☐ other

89. What is the way to pour out your troubles? [single choice] *

○ Never pour out your troubles to anyone

- ○ Only pour out your troubles to one or two persons who are very close to you
- ○ You would like to speak up your trouble If a friend asked you initiatively
- ○ You would like to speak up your trouble initiatively to gain support and understanding

90. What is the way to get help when you are in trouble? [single choice] *

- ○ To live on yourself, never ask anyone for help
- ○ Rarely ask for help
- ○ Sometimes ask for help
- ○ Always ask your family, friends and organizations for help

91. For the activities of organizations (such as party organizations, religious organizations, labor unions, students' unions, etc.), you… [single choice] *

- ○ never attend
- ○ participate occasionally
- ○ participate often
- ○ participate initiatively and actively

92. Your social support during the epidemic… [additional single choice] *

- ○ significantly increase
- ○ increase
- ○ have no change
- ○ decrease
- ○ significantly decrease

93. When do you feel you received the most social support during the domestic COVID-19 epidemic? [additional single choice] *

○ At the beginning

○ At the peak

○ At current stable period

94. When do you feel you received the least social support during the domestic COVID-19 epidemic? [additional single choice] *

○ At the beginning

○ At the peak

○ At current stable period

Educational and Living Conditions during COVID-19 Epidemic

95. How do you feel about the quality of your life? [single choice] *

○ Good

○ No change

○ Poor

96. Do you pay attention to COVID-19 epidemic? [single choice] *

○ Always

○ Usually

○ Sometimes

○ Almost never

97. What are the main sources which you usually receive the COVID-19 epidemic information from? [single choice] *

○ Wechat public accounts

○ Wechat friend circles

○ MicroBlogs

○ Short videoes

○ Official channels

○ Others

98. Have you ever gone to COVID-19 epidemic area (Hubei province)? [single choice] *

○ Yes

○ No

99. Is your hometown close to the COVID-19 epidemic area (The city of Nanyang, Xinyang or Zhumadian in Henan province)? [single choice] *

○ Yes

○ No

100. Have you or your family (who lived with you) been in contact with high-risk (confirmed or suspected) groups? [single choice] *

○ Yes

○ No

101. Are there any confirmed or suspected cases in your family? [single choice] *

○ Yes

○ No

102. Are there any confirmed or suspected cases among your relatives or friends? [single choice] *

○ Yes

○ No

103. Are there any confirmed or suspected cases in your community or village? [single choice] *

○ Yes

○ No

104. Have you ever had symptoms of fever? [single choice] *

○ Yes

○ No

105. Have other members of your family who live with you ever had a fever? [single choice] *

○ Yes

○ No

106. What is the impact of COVID-19 on your family's income? [single choice] *

○ Higher

○ As usual

○ Lower

107. Have you ever felt anxious about the risk of getting COVID-19 during the epidemic for yourself or your family?   [single choice] *

- ○ Not at all
- ○ Occasionally
- ○ Often

108. How often do you exercise during the epidemic? [single choice] *

- ○ Occasionally
- ○ Sometimes
- ○ Often

109. What is the change of your weight during the COVID-19 outbreak? [single choice] *

- ○ Weight gain
- ○ No change
- ○ Weight loss

110. How do you feel about your physical health condition during the COVID-19 epidemic? [single choice] *

- ○ Very good
- ○ Good
- ○ No change
- ○ Poor
- ○ Very poor

111. Do you have a regular schedule during COVID-19 epidemic? [single choice] *

- ○ Very regular

○ Regularly most of the time

○ Not regular

○ Very irregular

112. Your average time of leisure per day during the epidemic was... [single choice] *

○ within 1h

○ 1-3h

○ 3-6h

○ more than 6h

113. What are your ways of entertainments? [multiple choice] *

□ Play the game

□ View TikTok

□ View MicroBlog

□ Watch TV

□ Keep pets and plants

□ Do exercise

□ Baking and cooking

□ others

114. How do you get along with your family at home? [single choice] *

○ Obviously better

○ Better

- ○ No changes
- ○ Worse
- ○ Obviously worse

115. What is your study status during the epidemic? [single choice] *

- ○ Be proactive, and do it well
- ○ Make a plan, but can't carry it out
- ○ Easily disturbed, and unable to concentrate on study

116. How satisfied are you with the current online teaching? [single choice] *

- ○ Very satisfactory
- ○ Satisfactory
- ○ Average
- ○ Unsatisfactory

117. Have you adapted to the current online learning? [single choice] *

- ○ Completely
- ○ Almost
- ○ Not very well
- ○ Not at all

118. How about your mental state during online learning at home amid the COVID-19 epidemic? [single choice] *

- ○ Better

○ As usual

○ Worse

119. How about the influence of the learning condition and environment at home on your learning enthusiasm? [single choice] *

○ Very large

○ Large

○ A bit

○ A little

120. How about your study condition and environment at home? [single choice] *

○ Very good

○ Good

○ Common

○ Poor

121. What factors do you think can affect your study? [Multiple choice] *

○ The network is unstable

○ Family members

○ Learning condition and environment

○ others

122. How about your interest in studying at home during the epidemic? [single choice] *
(1~5 denotes degree value)

○ 1 ○ 2 ○ 3 ○ 4 ○ 5

123. What are the disadvantages of online learning? [Multiple choice] *

○ High requirement for students' self-discipline

○ The problems of network equipment signal and platform service

○ The teachers and students cannot communicate in a timely and effective manner

○ The teacher cannot supervise the students effectively during the online education

○ Others

124. What are the advantages of online learning? [Multiple choice] *

○ The teaching resources are abundant and shared

○ Teaching sessions can be recorded and played back

○ Travel costs of teachers and students can be saved (such as transportation fees)

○ The space-time boundary can be overcome

○ Others

125. What activities about online education do you think are conducive to learning? [Multiple choice] *

□ Live/taped course teaching

□ Completing the test assignments

□ Participating in online question-and-answer discussions

□ Participating in online mutual evaluation

□ Completing group tasks

□ Others

126. Which teaching methods do you prefer? [single choice] *

- O Classroom teaching
- O Online teaching
- O Combining online and offline teaching

127. During the COVID-19 epidemic, how do you study? [Multiple choice] *

- O Look up books
- O Search network resources independently
- O Learn the teaching resources provided by the school and teachers
- O Ask your classmates and friends
- O Others

128. What is your current study status? [single choice] *

- O Make study plans, and complete them adequately
- O Make study plans, and complete them mostly
- O Make study plans, but can’t keep up with the change
- O Have no plans, and study occasionally
- O Have no plans, and almost never study

129. Are you looking forward to back to school? [single choice] *

- O Expected
- O Don't expected

130. What are your reasons for looking forward to back to school? [multiple choice] *

- ☐ Miss friends (including boyfriend/girlfriend)

☐ Want living expenses

☐ Want to get into the normal study state

☐ Be away from family

☐ Others

131. What are your reasons for staying at home? [multiple choice] *

☐ Don't want to be separated from your family

☐ Haven't play enough

☐ Be used to the online course

☐ Save money

☐ Others

132. Has your psychological state been affected by the COVID-19 epidemic? [single choice] *

○ No

○ A little

○ Quite a bit

○ Very much

133. What is the impact of COVID-19 on your career planning? [single choice] *

○ Belief in medical profession has been firmed

○ No impact

○ Belief in medical profession has been shaken

○ Belief in medical profession has been changed resolutely

134. If you are a working medical staff in a hospital, would you like to be a front-line nurse? [single choice] *

○ Sure

○ May be

○ May be not.

○ Impossible

135. What kinds of psychological assistances would you like to receive in this COVID-19 epidemic? [multiple choice] *

☐ Medical knowledge

☐ Emotional regulation

☐ Behavioral guidance

☐ Family relationships

☐ Social support

☐ Counseling for psychological problems

☐ Emergency psychological assistance

☐ Others

Appendix

IES-R Scoring

**Options**	Not at all	A little bit	Moderately	Quite a bit	Extremely
**Scores**	0	1	2	3	4

Intrusion subscale contains items of IES-R 1, 2, 3, 6, 9, 14, 16, 20.

Avoidance subscale contains items of IES-R 5, 7, 8, 11, 12, 13, 17, 22.

Hyperarousal subscale contains items of IES-R 4, 10, 15, 18, 19, 21.

The severity of post-traumatic stress disorder (PTSD) symptom is evaluated by sum score of intrusion and avoidance subscales.

IES-R Severity Rating

**Rating**	Normal	Mild	Moderate	Severe
**Scores**	0-8	9-25	26-43	44-64

Reference

1. Christianson S, Marren J. The Impact of Event Scale - Revised (IES-R). Medsurg Nurs. 2012, 21(5):321-322. PMID: 23243796. doi:10.1007/s11800-008-0132-2

DASS-21 Scoring

Options	Scores
Do not apply to me at all	0
Apply to me to some degree or some of the time	1
Apply to me a considerable degree or a good part of the time	2
Apply to me very much or most of the time	3

1) Questions numbered DASS 3, 5, 10, 13, 16, 17, 21 belong to **Depression** subscale.

2) Questions numbered DASS 2, 4, 7, 9, 15, 19, 20 belong to **Anxiety** subscale.

3) Questions numbered DASS 1, 6, 8, 11, 12, 14, 18 belong to **Stress** Subscale.

The final score of each subscale (Depression, Anxiety and Stress) is equal to sum score of its items then multiply by two (×2), due to the DASS 21 is a short form version of the DASS (the Long Form has 42 items).

Refer to the number in the table below to determine how mild or serious each condition may be.

DASS Severity Rating (sum score ×2)

Rating	Depression score	Anxiety score	Stress score
Normal	0-9	0-7	0-14
Mild	10-13	8-9	15-18
Moderate	14-20	10-14	19-25
Severe	21-27	15-19	26-33
Extremely Severe	28-42	20-42	34-42

References

1. Lovibond PF, Lovibond SH. The structure of negative emotional states: comparison of the Depression Anxiety Stress Scales (DASS) with the Beck Depression and Anxiety Inventories. Behav Res Ther. 1995, 33(3):335-343. PMID: 7726811. doi:10.1016/0005-7967(94)00075-u

2. Henry JD, Crawford JR. The short-form version of the Depression Anxiety Stress Scales (DASS-21): construct validity and normative data in a large non-clinical sample. Br J Clin Psychol. 2005, 44(Pt 2):227-239. PMID: 16004657. doi:10.1348/014466505X29657

PSQI Scoring

Options	Scores
Not during the past month/Very good	0
Less than once a week/Fairly good	1
Once or twice a week/Fairly bad	2
Three or more times week/Very bad	3

1) **Component 1 (sleep quality)**, the score of PSQI 6.

2) **Component 2 (sleep latency)**, the score of PSQI 2 [≤15min (0); 16-30min (1), 31-60min (2), 60min (3)] + the score of PSQI 5a, if sum is equal 0=0, 1-2=1, 3-4=2, 5-6=3.

3) **Component 3 (sleep duration)**, the score of PSQI 4 (>7h=0, 6-7h=1, 5-6h=2, <5h=3).

4) **Component 4 (sleep efficiency)**, actual sleep hours (PSQI 4) / total hours in bed ×100% (>85%=0, 75%-84%=1, 65%-74%=2, <65%=3)

5) **Component 5 (sleep disturbance)**, the sum of Scores PSQI 5b to 5j (0=0, 1-9=1, 10-18=2, 19-27=3).

6) **Component 6 (use of sleeping medication)**, the score of PSQI 7.

7) **Component 7 (daytime dysfunction)**, the sum score of PSQI 8 and 9 (0=0, 1-2=1, 3-4=2, 5-6=3).

PSQI score is equal to sum of the seven component scores.

PSQI Severity Rating

PSQI	Sleep quality
0-5	Normal
6-10	Mild insomnia
11-15	Moderate insomnia
16-21	Severe insomnia

Reference

1. Buysse DJ, Reynolds CF 3rd, Monk TH, Berman SR, Kupfer DJ. The Pittsburgh Sleep Quality Index: a new instrument for psychiatric practice and research. Psychiatry Res. 1989, 28(2):193-213. PMID: 2748771. doi:10.1016/0165-1781(89)90047-4
